# Supplementary figures and images for: Actomyosin contractility is a potent suppressor of mesoderm induction by human pluripotent stem cells
Source: J Cell Biol. 2026 Apr 24;225(5):e202507103. doi: 10.1083/jcb.202507103 (PMC13108843; doi:10.1083/jcb.202507103)

Source data - Figure 2 C

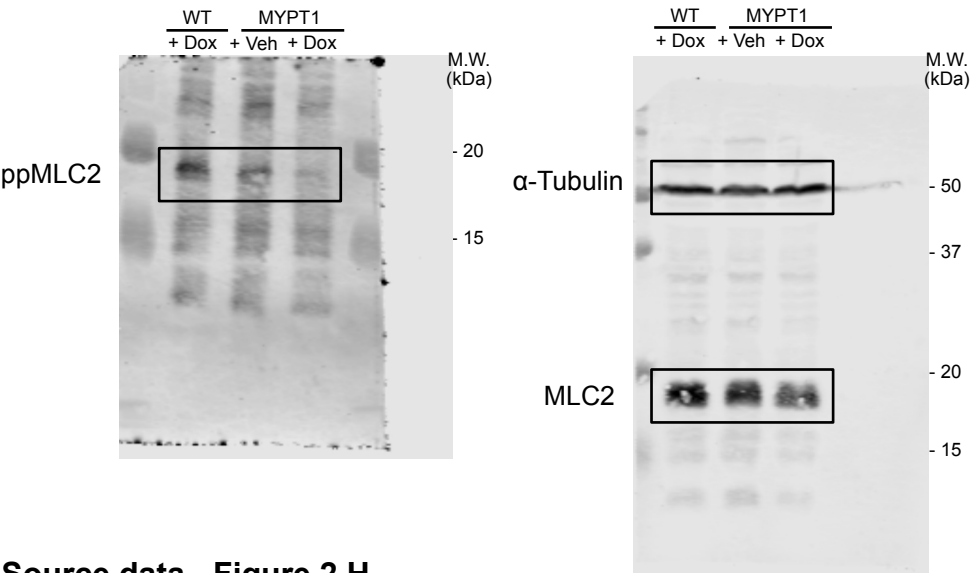

Source data - Figure 2 H

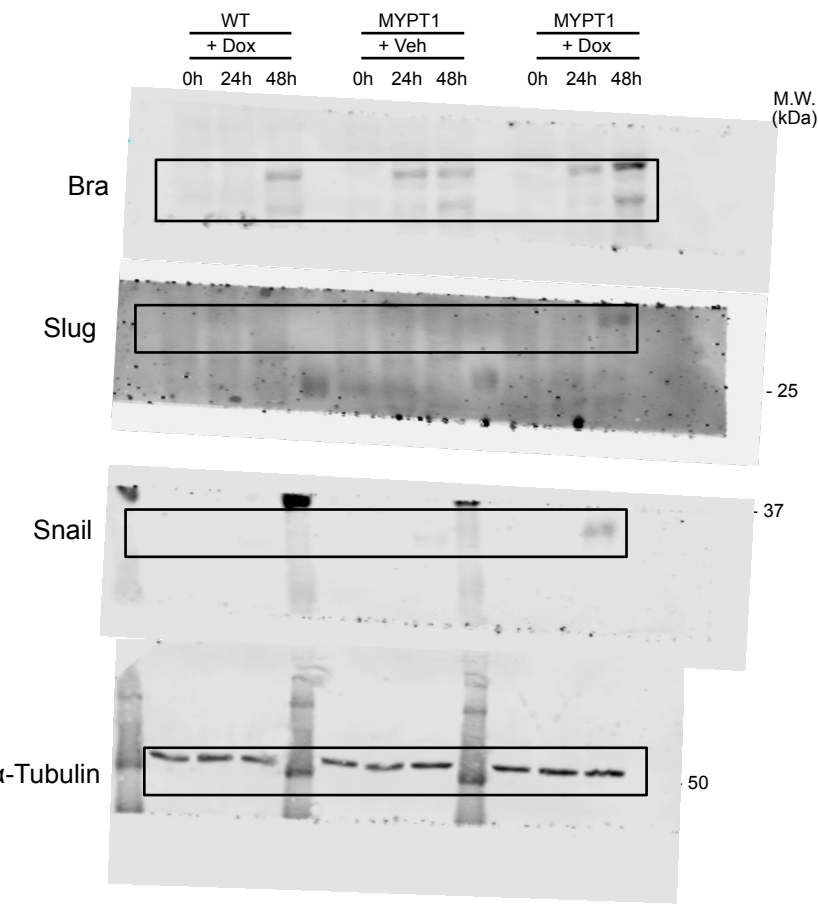

Source data - Figure 2 J

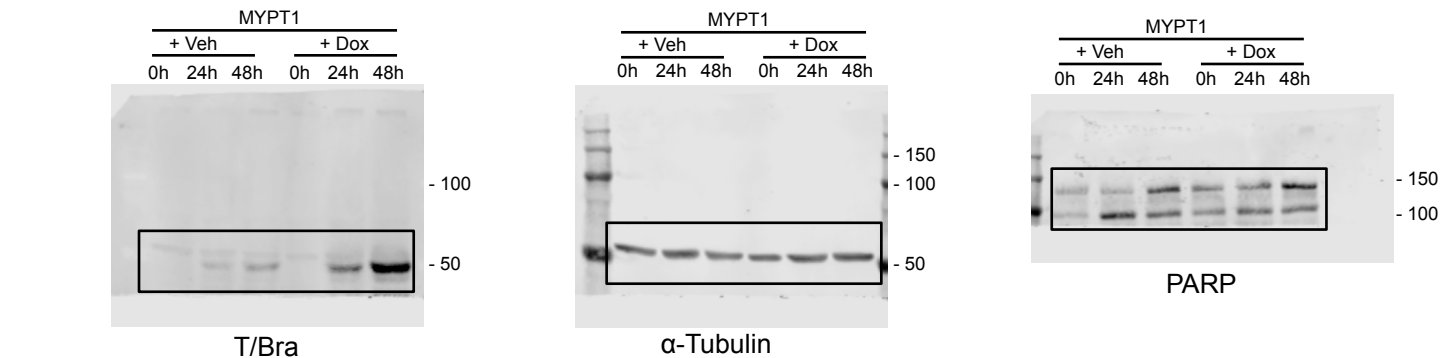

Supplement: SourceData F2 — is the source file for Fig. 2. [file jcb_202507103_sourcedataf2.pdf]

Source data - Figure 3B

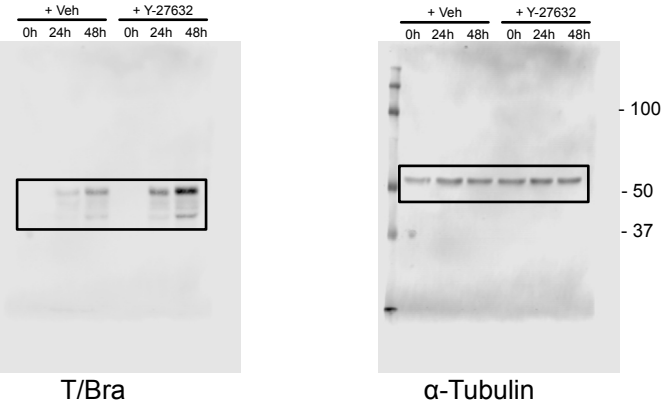

Source data - Figure 3O

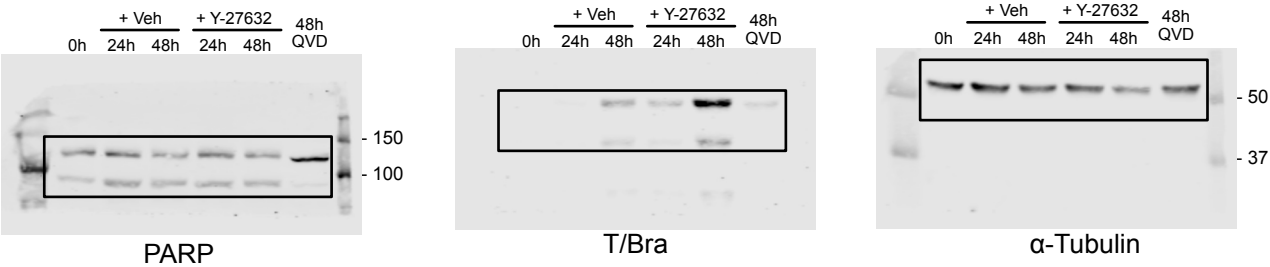

Source data - Figure 3R

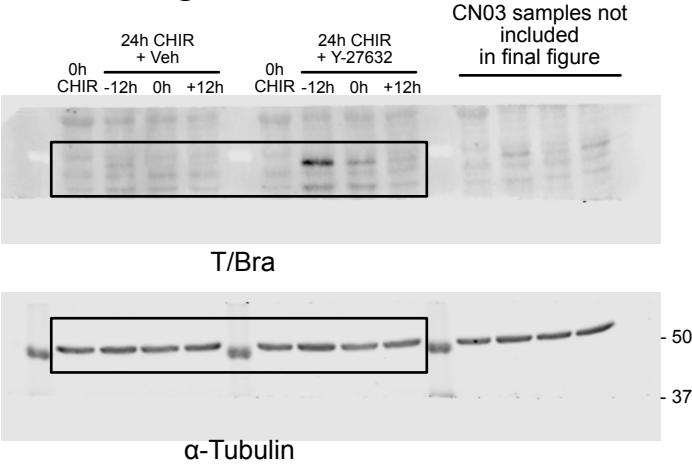

Supplement: SourceData F3 — is the source file for Fig. 3. [file jcb_202507103_sourcedataf3.pdf]

Source data - Figure 4B

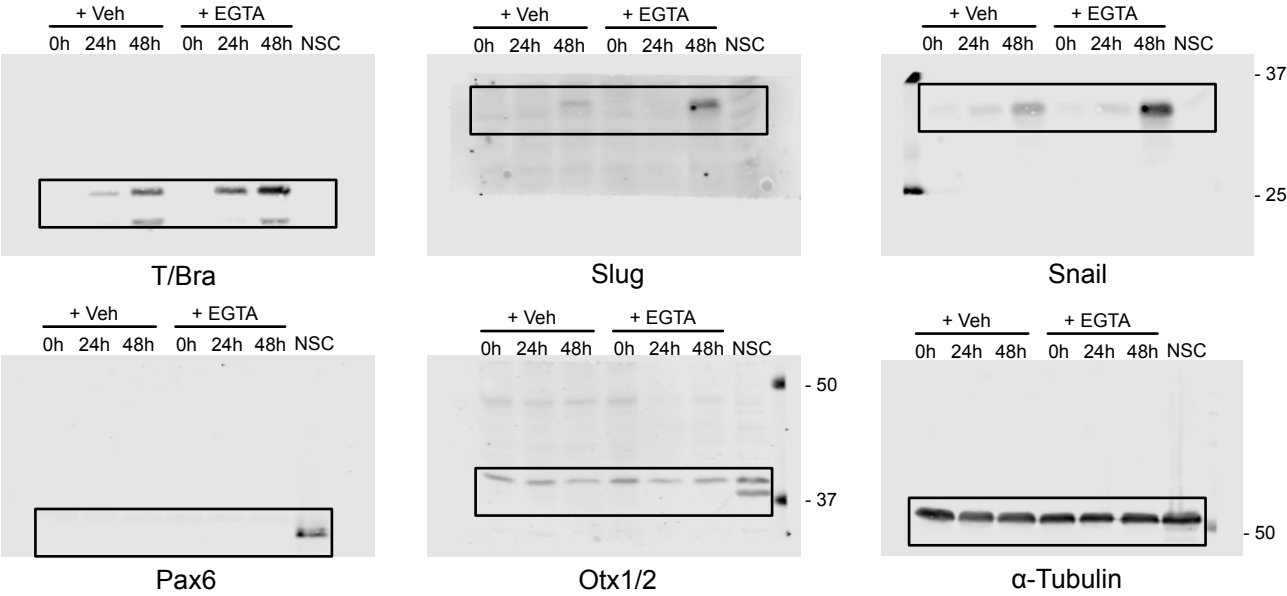

Source data - Figure 4H

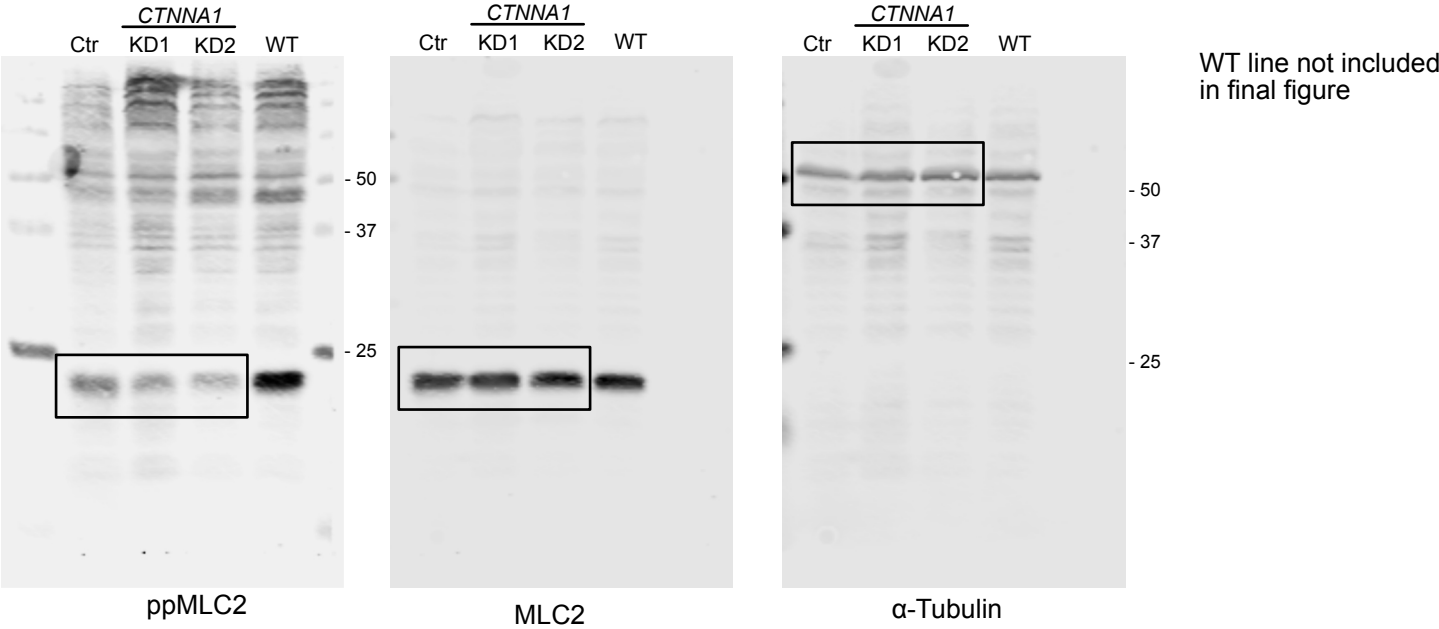

Source data - Figure 4H

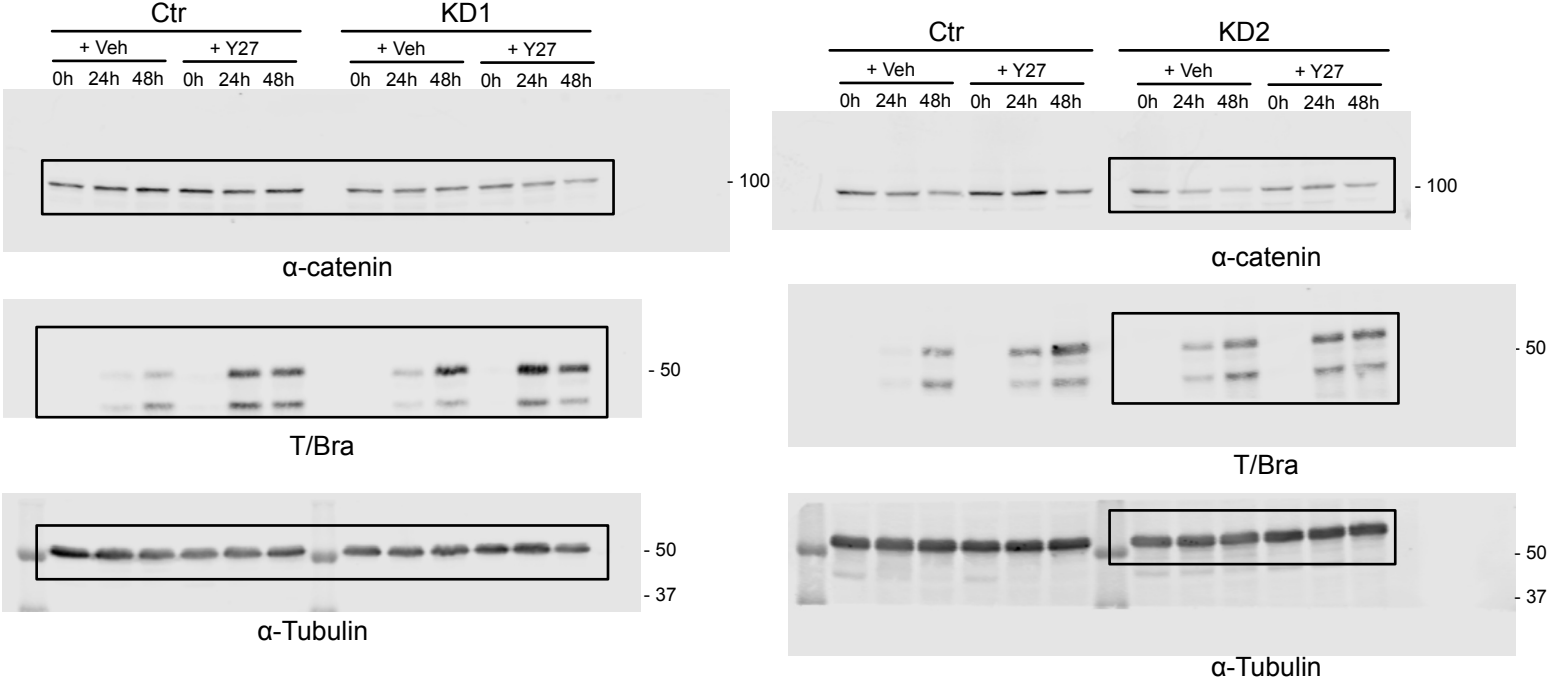

Supplement: SourceData F4 — is the source file for Fig. 4. [file jcb_202507103_sourcedataf4.pdf]

Source data - Figure 6E

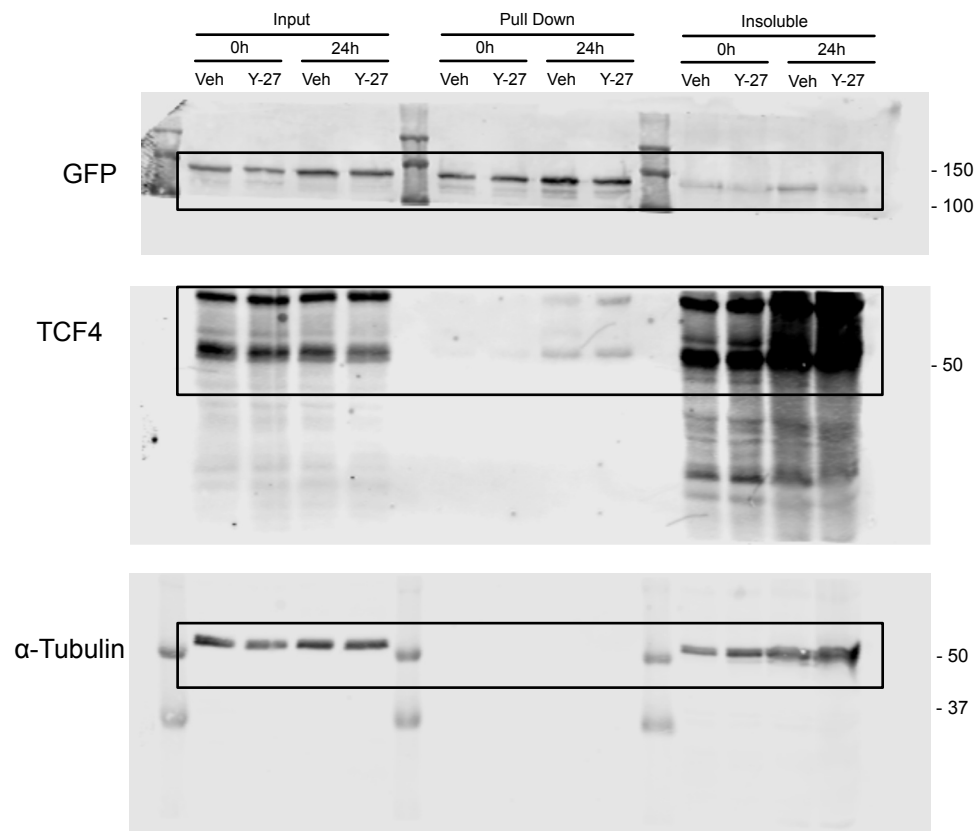

Supplement: SourceData F6 — is the source file for Fig. 6. [file jcb_202507103_sourcedataf6.pdf]

Source data - Supplementary Figure 4C

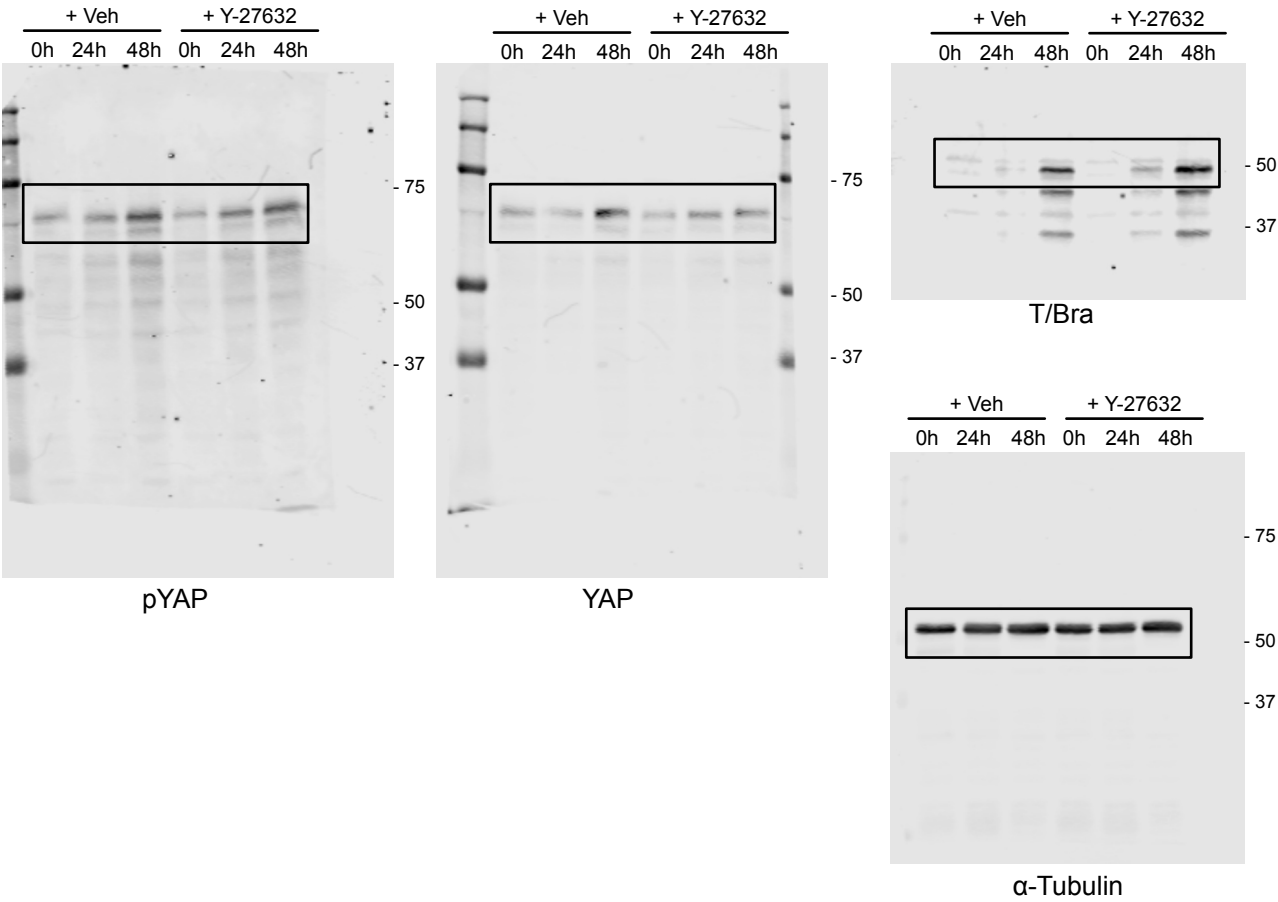

Supplement: SourceData FS4 — is the source file for Fig. S4. [file jcb_202507103_sourcedatafs4.pdf]

Source data - Supplementary Figure 5F

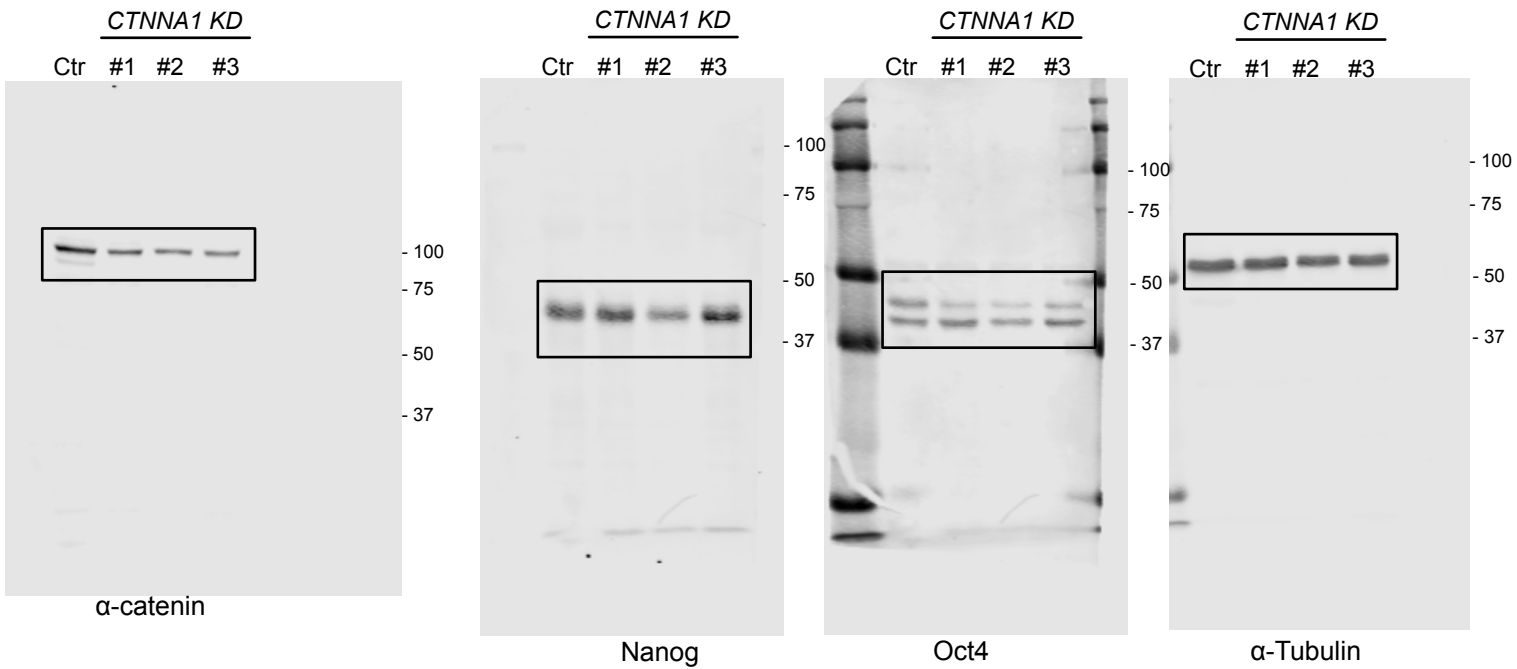

Supplement: SourceData FS5 — is the source file for Fig. S5. [file jcb_202507103_sourcedatafs5.pdf]

Source data - Supplementary Figure 6A

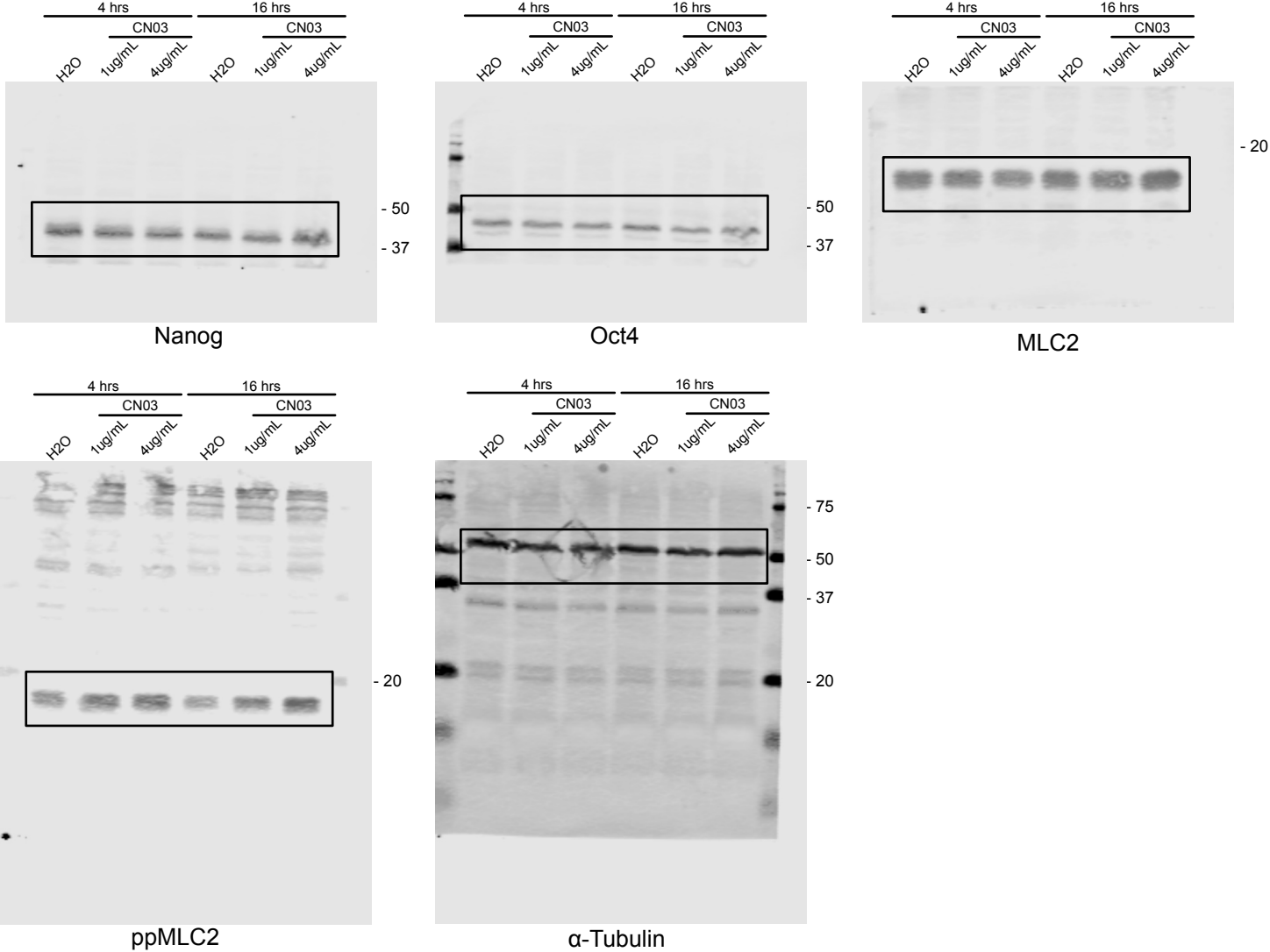

Source data - Supplementary Figure 6E

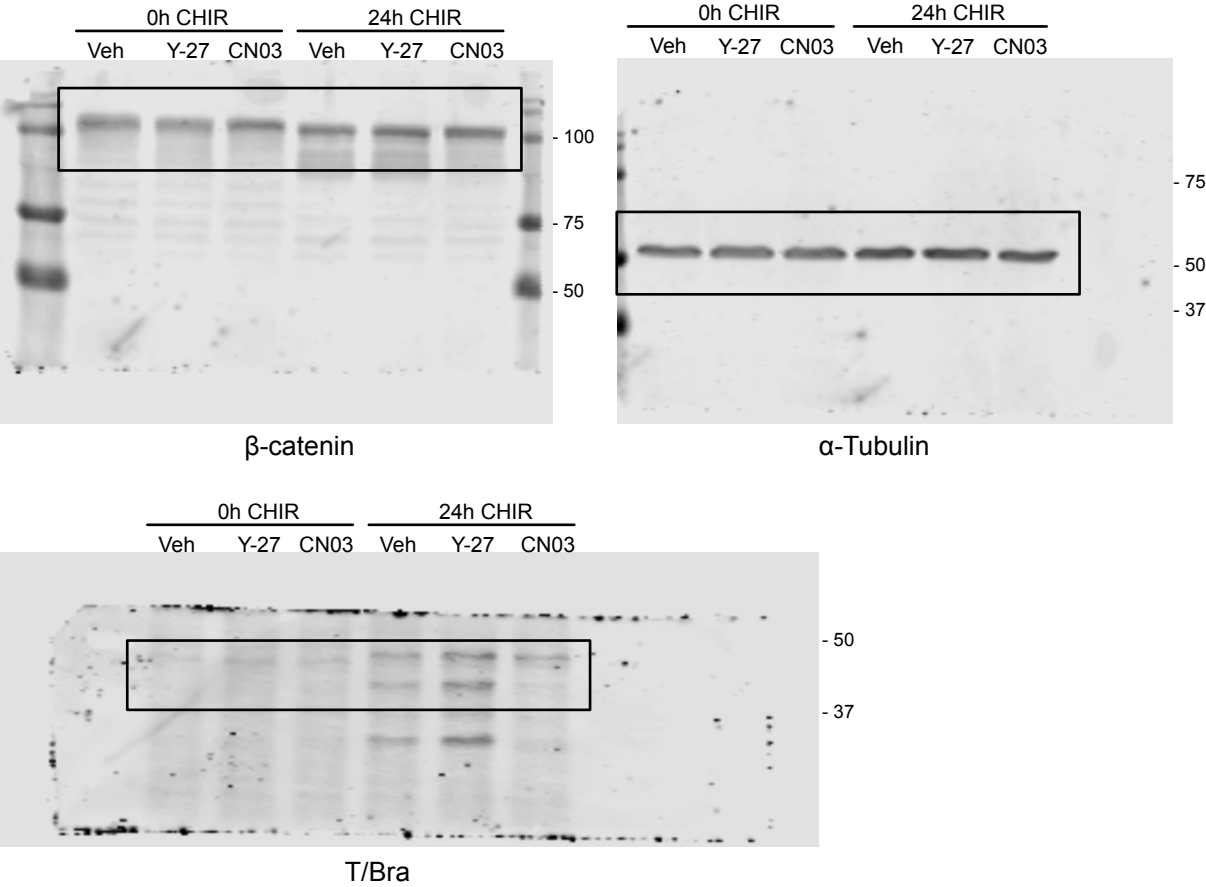

Supplement: SourceData FS6 — is the source file for Fig. S6. [file jcb_202507103_sourcedatafs6.pdf]
