# Supplementary material for: Actomyosin contractility is a potent suppressor of mesoderm induction by human pluripotent stem cells
Source: J Cell Biol. 2026 Apr 24;225(5):e202507103. doi: 10.1083/jcb.202507103 (PMC13108843; doi:10.1083/jcb.202507103)

Source data - Supplementary Figure 2L

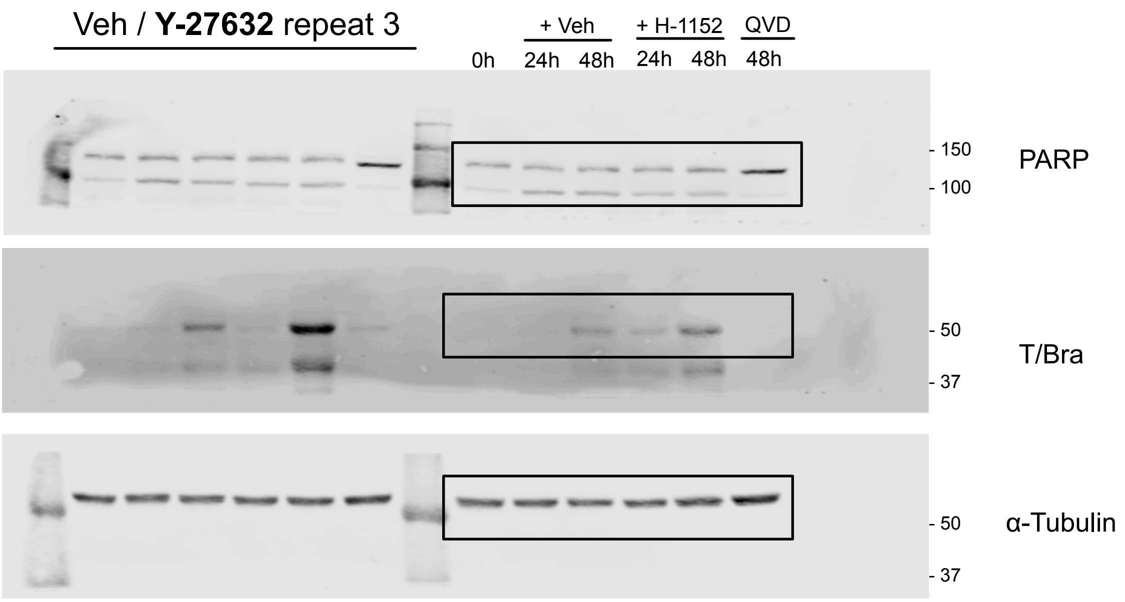

Source data - Supplementary Figure 2N

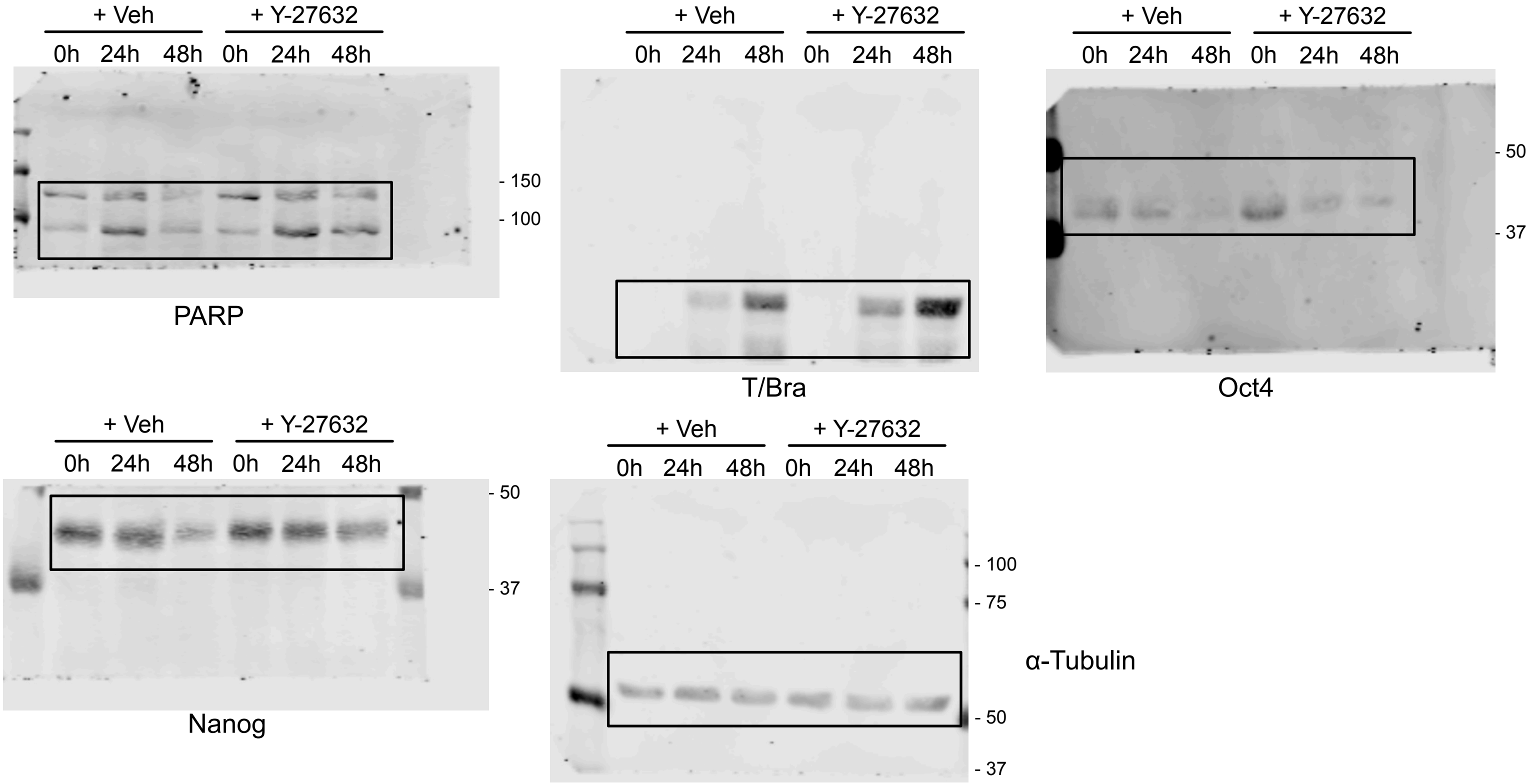

Source data - Supplementary Figure 2R

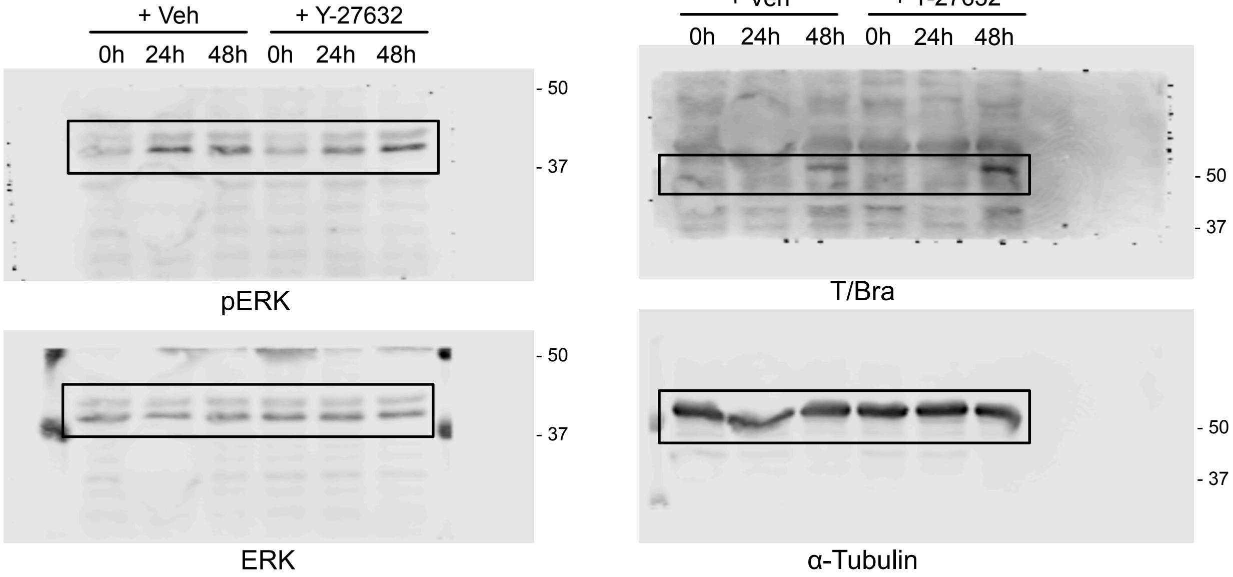

Supplement: SourceData FS2 — is the source file for Fig. S2. [file jcb_202507103_sourcedatafs2.pdf]
